# Risk of Anaplastic Large-Cell Lymphoma (ALCL) in Cases of Late Seroma Formation After Breast Implant Insertion

**Published:** 2021-03-08

**Authors:** Kun Hwang, Hun Kim, Hyung Mook Kim, Joo Ho Kim

**Affiliations:** ^a^Department of Plastic Surgery, Inha University School of Medicine, Incheon, Korea; ^b^Department of Plastic Surgery, Inha University Hospital, Incheon, Korea

**Keywords:** lymphoma, large-cell, anaplastic, seroma, breast implants, meta-analysis, odds ratio

## Abstract

**Purpose:** The aim of this study was to determine the odds ratio of anaplastic large-cell lymphoma in late seroma formation. **Methods:** In a PubMed search, 415 articles were found using the terms “breast implant AND seroma” (n = 232), “breast implant AND effusion” (n = 42), and “anaplastic large cell lymphoma AND breast (n = 141). Sixty-seven abstracts were read, and 27 full articles were reviewed. **Results:** Three articles reported the incidence of late seroma in breast implants, with a total of 75 seromas out of 48,211 implants (0.16%). One article reported 48 cases of non-Hodgkin lymphoma from 43,537 implants (0.11%). Another article reported that 11 patients had anaplastic large-cell lymphoma among 389 primary lymphoma of the breast (2.83%). Two articles reported 143 seromas out of 236 anaplastic large-cell lymphomas (60.59%). The risk of anaplastic large-cell lymphoma was significantly higher in the patients having late seroma than those without seroma (odds ratio = 998.93; 95% confidence interval, 768.90-1297.78; *P* < .001). The incidence of anaplastic large-cell lymphoma in seroma was calculated by dividing the number of anaplastic large cell lymphomas with seroma (n = 143) by total seroma (N = 11,843), which resulted in an incidence of 1.21%. The expected incidence of anaplastic large-cell lymphoma in seroma was 1.21%. **Conclusion:** If late seroma develops after breast implant insertion, ultrasonography-guided aspiration should be performed, with enzyme-linked-immunosorbent serologic assay for CD30.

Breast implant–associated anaplastic large-cell lymphoma (ALCL) has recently come to the attention of researchers and clinicians. It most frequently presents as an effusion-associated fibrous capsule surrounding an implant but can present as a mass in some cases.^1^ According to the American Cancer Society, approximately 2% of lymphomas can be classified as ALCL.^2^ Recently, several review articles about ALCL have been published, but very few studies have focused on the association of ALCL with late seroma.^1^^,^^3^^,^^4^

The aim of this study was to determine the odds ratio (OR) of late seroma formation for ALCL.

## METHODS

**Figure 1:**
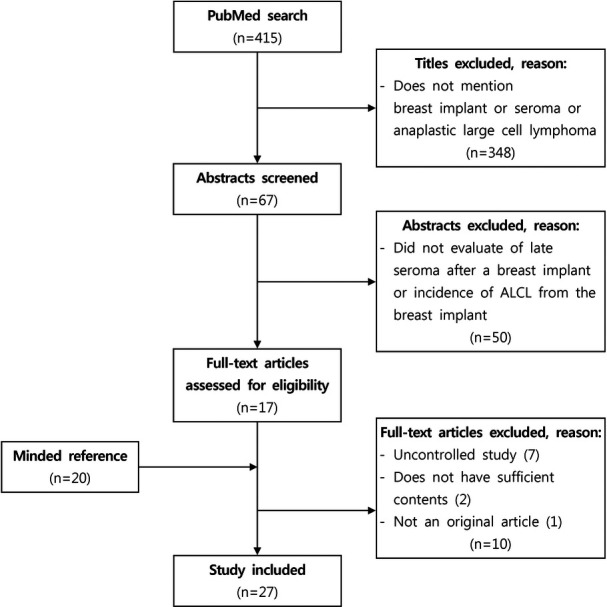
PRISMA flowchart, showing the selection process of the articles included in this study. ALCL indicates anaplastic large-cell lymphoma.

In a PubMed search, 415 articles were found using the terms “breast implant AND seroma” (n = 232), “breast implant AND effusion” (n = 42), and “anaplastic large cell lymphoma AND breast” (n = 141). Among the 415 titles, 348 were excluded while 67 abstracts met our inclusion criteria (“breast implant” or “seroma” or anaplastic large cell lymphoma” appeared in the title). Studies that did not include an evaluation of the incidence of late seroma or ALCL after breast implantation were excluded. Using these exclusion criteria, 50 abstracts were excluded and 17 full-text articles that evaluated the incidence of late seroma or ALCL in patients who underwent breast implantation were reviewed. Of these 17 articles, 10 were excluded because they were uncontrolled studies (7 articles), did not have sufficient content (2 articles), or were not an original article (1 article), while 20 articles were added from the reference list of the articles identified in the searches. Ultimately, 27 articles were analyzed (Fig 1).^4^^-^30

No restrictions on language and publication form were imposed. However, the full-text articles were mostly in English. All articles were read by 2 independent reviewers who extracted data from the articles.

To determine the relationship of seroma with ALCL, the data were summarized and the OR for ALCL between patients who did and did not have seroma was calculated. Weighted mean differences and 95% confidence intervals (CIs) were also calculated.

A statistical analysis was performed with Review Manager (The Nordic Cochrane Centre). If the exact number of the cases was not specified in the article, it was calculated from the reported percentage of incidence.

## RESULTS

Among the 7 articles analyzed, 6 articles had follow-up length.^4^^,^^6^^-^9^,^^30^ The average length of follow-up was 8.9 ± 5.3 years (range, 0.5-20 years).

**Figure 2:**
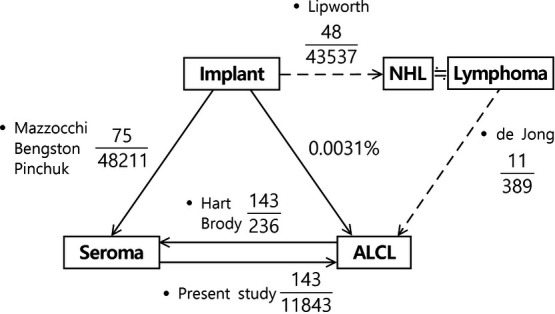
Relationships among breast implant, seroma, and ALCL. Seroma was found in 0.16% of patients with implants. ALCL was found in 0.0031% of patients with implants. Furthermore, 60.59% of ALCL patients had seroma. The expected incidence of ALCL in patients with seroma patients was 1.21%. ALCL indicates anaplastic large-cell lymphoma.

1. *Incidence of late seroma in patients who underwent breast implantation*. Three articles reported the incidence of late seroma in patients who underwent breast implantation, with a total of 75 seromas in 48,211 implants (0.16%) (Table 1).^5^^-^7
2. *Incidence of ALCL in patients who underwent breast implantation*. One review article reported 48 cases of non-Hodgkin lymphoma (NHL) in 43,537 patients who underwent breast implantation (0.11%) (Table 2, *top*).^8^ Another article stated that 11 patients had ALCL among 389 patients with primary lymphoma of the breast (2.83%) (Table 2, *bottom*).^9^
  Primary lymphomas of the breast are almost always NHL, and only 29 cases of Hodgkin lymphoma were reported.^10^^-^29 Thereafter, the incidence of ALCL among all breast implants was calculated by multiplying the incidence of NHL in breast implants by the incidence of ALCL in patients with breast lymphoma (48/43,537 × 11/389 × 100 = 0.0031%).
3. *Frequency of seroma in ALCL patients*. Two articles reported the frequency of seroma in ALCL patients, with a total of 143 seromas in 236 ALCLs (60.59%) (Table 3).^4^^-^30
4. *Odds ratio and risk ratio of ALCL in patients with late seroma*. Using the aforementioned incidence values, the OR and risk ratio (RR) were calculated (Fig 2, Table 2). The number of ALCLs in patients without seroma (n = 93) was calculated by subtracting the number of ALCLs in patients with seroma (n = 143) from the total number of ALCLs (N = 236). The total number of implant cases was estimated using the proportional expression of the incidence (0.0031%) of ALCL among breast implants (236 × 0.000031 = 7,612,903). The total number of seroma cases was estimated using the proportional expression of the incidence (0.16%) of late seroma among patients with breast implants (7,612,903 × 75/48,211 = 11,843). Then, the remaining blanks were filled by subtracting or adding the known numbers and the OR and RR were calculated.
  The risk of ALCL was significantly higher in patients with late seroma than in patients without seroma (OR = 998.93; 95% CI, 768.90-1297.78; *P* < .001; and RR = 986.88; 95% CI, 760.58-1280.53; *P* < .001).
5. *Incidence of ALCL in patients with seroma*. The incidence of ALCL in patients with seroma was calculated by dividing the number of cases of ALCL in patients with seroma (n = 143) by the total number of cases of seroma (N = 11,843), resulting in an incidence of 1.21% (Table 4).

## DISCUSSION

Breast implant–associated ALCL is a rare type of lymphoma that was first described in 1997.^31^ In this review, the term “late seroma” encompassed any fluid accumulation that occurred more than 1 year after surgery in patients with breast implants, following the definition presented by Bengtson et al.^6^

In total, the US Food and Drug Administration (FDA) is aware of approximately 733 case reports of ALCL in women with breast implants worldwide. The total number of implants worldwide is estimated to be between 5 million and 10 million.^32^ On the basis of these figures, the frequency of breast implant–associated ALCL can be estimated as 12 per million (1 case/86,000 population) to 453 per million (1 case/2207 population).

As of January 5, 2020, the FDA has received a total of 733 medical device reports of breast implant–associated ALCL, including 36 deaths, of which 17 reports included information on the implant surface. Of the 733 total unique cases of breast implant–associated ALCL reported to the FDA, 496 cases were reported to have textured implants and 209 cases did not specify the implant surface. Of the 36 total patient deaths reported to the FDA, 16 cases reported textured implants and 19 cases did not contain information on the implant surface.^33^

To investigate the association of silicone breast implants with immunological abnormalities, Karlson et al^34^ selected 200 women who had been exposed to silicone breast implants and 500 age-matched, nonexposed women as controls. No increased frequency of any immunological abnormalities was found in women exposed to silicone breast implants, except for anti-ssDNA.^34^

Despite the apparent strong association between breast implants and ALCL, which typically surrounds the implants, suggesting an etiologic relationship, the cause of breast implant–associated ALCL remains unknown.^1^

Since concerns have been raised regarding seroma in breast implants, we searched the literature and found 3 articles that could be used to calculate the incidence.^5^^-^7

For ALCL and breast implants, we could not find articles containing many clinical cases. In the Lipworth et al^8^ review, 48 cases of NHL were found in 43,537 breast implants (Table 2, *top*). de Jong et al^9^ reported that 11 patients had ALCL among 389 patients with primary lymphoma of the breast (2.83%) (Table 2, *bottom*). Primary lymphomas of the breast are almost always NHL, and only 29 cases of Hodgkin lymphoma have been reported.^10^^-^29 Therefore, the incidence of ALCL in breast implants was calculated by multiplying the incidence of NHL in breast implants by the incidence of ALCL in patients with breast lymphoma (0.0031%).

It is interesting that late seromas had not been mentioned in the era of smooth implants; instead, late seromas were only reported after the introduction of textured implants.^5^

The studies used for our analysis included confirmed ALCL cases. In our present review, seroma was reported in 0.16% of patients with implants and ALCL was found in 0.0031% of patients with implants. Moreover, 60.59% of patients with ALCL had seroma. The expected incidence of ALCL in patients with seroma was 1.21% (Fig 2).

The risk of ALCL was significantly higher in patients with late seroma than in those without seroma (OR = 998.93).

Clemens et al^35^ emphasized that is important to differentiate between breast implant–associated ALCL and late seroma, as they are distinct unrelated processes, despite the common misperception that they are on a continuum, with one leading into the other. Benign fluid collections are not precursors to the development of breast implant–associated ALCL, and, to date, no cases have been reported of recurrent benign seromas converting to a CD30-positive effusion.^35^

Recently, Hanson et al^36^ utilized in situ enzyme-linked immunosorbent assay (ELISA) to screen 9 patients with breast implant–associated ALCL and demonstrated that CD30 was detected in all breast implant–associated effusions at full and all serial concentrations. However, the breast implant–associated ALCL serum specimens and all control specimens showed negative findings.^36^

On the basis of the aforementioned studies, it is recommended to test all delayed seromas that are not otherwise readily explainable with CD30 immunohistochemistry.

We suggest the following protocol for managing patients presenting with a late seroma:

1. Ultrasonography-guided aspiration is performed with ELISA for CD30.
2. In recurrent seroma with persistently negative markers, the implant is removed.
3. If an abnormal capsule is seen, it is biopsied and sent for a pathological examination.
4. A smooth-surfaced implant is inserted if necessary.

## Figures and Tables

**Table 1:** Incidence of late seroma in breast implant

Author	Patients with seroma	Breast implants	Incidence (%)
Mazzocchi et al (2010)^5^	8	435	1.84
Bengtson et al (2011)^6^	62	47,208	0.13
Pinchuk and Tymofii (2011)^7^	5	568	0.88
Total	75	48,211	0.16

**Table 2:** Incidence of non-Hodgkin lymphoma in breast implant and anaplastic large-cell lymphoma in breast lymphoma*

	NHL patients	Breast implants	Incidence (%)
Lipworth et al (2009)^8^	48	43,537	0.11
	**ALCL patients**	**Breast lymphoma**	**Incidence (%)**
de Jong et al (2008)^9^	11	389	2.83

*NHL indicates non-Hodgkin lymphoma; ALCL, anaplastic large-cell lymphoma.

**Table 3:** Seroma and anaplastic large-cell lymphoma*

Author	Patients with seroma	ALCL implants	Incidence (%)
Hart et al (2014)^4^	39	63	61.90
Brody et al (2015)^30^	104	173	60.12
Total	143	236	60.59

*ALCL indicates anaplastic large-cell lymphoma.

**Table 4:** Odds ratio and risk ratio of anaplastic large-cell lymphoma in late seroma*

	ALCL		Total	OR (95% CI)	RR (95% CI)
	+	−			
Seroma					
+	143	11,700	11,843	998.93 (768.90-1297.78)	986.88 (760.58-1280.53)
−	93	7,600,967	7,601,060		
Total	236	7,612,967	7,612,903	*P* < .001	*P* < .001

*ALCL indicates anaplastic large-cell lymphoma; OR, odds ratio; CI, confidence interval; RR, relative risk.
